# The application of rigid and flexible mediastinoscopy in esophagectomy: our experience and a new technology

**DOI:** 10.1186/s12957-021-02352-w

**Published:** 2021-08-07

**Authors:** Chun-Li Wu, Bo Dong, Bin Wu, Shi-Hao Li, Yu Qi

**Affiliations:** Department of Thoracic Surgery, The First Affiliated Hospital of Zhengzhou University, No. 1 Jianshe East Road, Zhengzhou, 450052 Henan Province China

**Keywords:** Flexible mediastinoscopy, Mediastinoscopy-assisted esophagectomy, MAE, Flexible endoscopy, Early esophageal cancer

## Abstract

**Background:**

To avoid the inconvenience of triangulation among various rigid operating instruments in mediastinoscopy-assisted esophagectomy, we invented a new technique: used a flexible endoscope to mobilize thoracic esophagus and dissected mediastinal lymph nodes through the left cervical incision. This technology has not been reported so far. In this study, we introduce our long-term experience and demonstrate this new technique.

**Methods:**

Twenty-nine patients with early esophageal cancer underwent mediastinoscopy-assisted esophagectomy in our hospital from June 2018 to September 2020. Among them, 12 patients used flexible mediastinoscopy, and 17 patients used conventional rigid mediastinoscopy and instruments to observe their therapeutic effect.

**Results:**

There were no significant differences between the two groups in gender, average age, body mass index, incidence of adverse reactions, bleeding volume, and postoperative hospital stay. The operation time of flexible mediastinoscopy group was significantly shorter than that of rigid mediastinoscopy group (192.9 ± 13.0 vs 246.8 ± 6.9 min, *p* < 0.01). The number of lymph nodes removed by flexible endoscopy was significantly more than that of rigid endoscopy (8.5 ± 0.6 vs 6.0 ± 0.3, *P* < 0.01). Postoperative follow-up was completed for all patients, and the average follow-up time was 11.6 ± 7.2 months. During the follow-up period, no recurrence or death was observed.

**Conclusions:**

Mediastinoscopy-assisted esophagectomy is an effective way to treat early esophageal cancer. The application of flexible mediastinoscopy provides more convenience and better stability. It can facilitate the operation of the surgeon and lymph node dissection, which proved to be a feasible technology.

## Introduction

Numerous literatures indicate that mediastinoscopy treatment of esophageal cancer is a feasible operation since mediastinoscopy-assisted esophagectomy (MAE) was proposed in 1990 [1–3]. MAE is an advanced surgical technique, but due to its rigid characteristics and the limitation of other rigid equipment (ultrasonic scalpel, suction device, etc.) required in a narrow space during the surgery, it is inconvenient and only used as an alternative to conventional surgery in early esophageal cancer patients with cardiopulmonary dysfunction. In addition, the almost horizontal angle has limitations in mediastinal lymphadenectomy.

In order to reduce the damage to the mediastinum caused by the equipment and its rigid characteristic, and perform lymphadenectomy more conveniently, we have developed a new surgical technique: flexible mediastinoscopy. The flexible endoscope is used for the operation of thoracic esophagus and lymphadenectomy. So far, there is no report on the clinical application of this technology.

## Methods

### Patients

From June 2018 to September 2020, 29 early esophageal cancer patients (Tis-T1) were incapable of endoscopic treatment due to extensive lesions or the deeper infiltration. So they underwent MAE in our hospital, and 12 patients among them underwent surgery with flexible mediastinoscopy. After explaining the difference between the two operations, the attending doctor determined the operating instruments according to the patients’ own choice.

All the patients came from the same outpatient department. Rigid mediastinoscopy, laparoscopy, and gastroesophageal anastomosis were performed by the same surgical team. Flexible mediastinoscopy was operated by experienced endoscopists under the guidance of thoracic surgeons. This study was approved by the Ethics Committee of the First Affiliated Hospital of Zhengzhou University.

### Surgical technique

According to standard surgical procedures, mediastinoscopy and laparoscopy were performed at the same time. The patients were placed in supine position, under general anesthesia with endotracheal intubation. Made an oblique incision along the sternocleidomastoid muscle; dissected cervical and thoracic esophagus; dissociated the stomach under laparoscopy; converged with the mediastinoscopy; made a tubular stomach; performed the gastroesophageal anastomosis in the neck. Mediastinal drainage tube and enteral nutrition tube were indwelled after operation. Mediastinoscopy was performed through a 5-cm long transverse incision on the left clavicle. After we fully dissociated and severed the cervical esophagus, the distal esophagus resection edge was connected to suture for traction to facilitate the anastomosis in the neck. Generally, the 3rd day after surgery, all the patients performed chest CT to see if there was severe lung infection or massive pleural effusion. When there was no chylous exudation and drainage volume < 50 ml/day, the mediastinal drainage tube was removed. Enteral nutrition was given on the 4th day after surgery. That is to say, the patients used a syringe to inject food into the gastrointestinal tract through a nasal feeding tube. After the nutrition tube was indwelling for a period of time, when the patients had upper gastrointestinal angiography and that showed no anastomotic stenosis or anastomotic leakage, the gastrointestinal nutrition tube was pulled out and the patients began to eat by mouth. Neither group of patients had undergone radiotherapy nor chemotherapy during treatment.

### Rigid mediastinoscopy

**Fig. 1:**
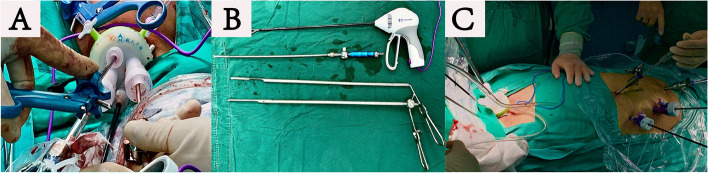
**A** The apparatus of rigid mediastinoscopy group was introduced into mediastinum cavity through a sealed lap-protector of the neck. **B** Required equipment of rigid mediastinoscopy group: LCS, three long retractors assisting in exposing the operating vision. **C** The surgical demonstration of rigid mediastinoscopy group

Made an oblique cervical incision. Exposed carotid artery and jugular vein. Exposed cervical esophagus carefully under direct vision to preserve the recurrent laryngeal nerve. Made a surgical space by blunt dissection with fingers. A sealed lap-protector was inserted into the cervical incision. CO2 gas was blown in to provide a stable surgical field (pressure 15 mmHg, flow 20 L/h), and then we carefully pushed the operating instrument into the upper mediastinum cavity along the left side of esophagus (Fig. 1A). Suction device was used to divide esophagus from trachea on the anterior side of the esophagus. Surgery on the neck required the cooperation of the surgeon, assistant, and another doctor holding the mediastinoscope. The assistant mainly used the long retractor to assist in exposing the operating vision (Fig. 1B). The cervical esophagus was pulled to the left side when isolating the thoracic esophagus on the right side. Dissecting the esophagus clockwise downwards, the left recurrent laryngeal nerve, posterior thoracic duct, and important vessels should be protected or ligated. The dissection was mainly assisted by the laparosonic coagulating shears (LCS) and duckbill pliers. The azygos arch and bronchial arteries were exposed by LCS at the right side of the esophagus. The anterior tracheoesophageal ligament was directly dissociated, and trachea bifurcation/hilar lymph nodes were visible. We separated the lymph nodes by blunt dissection. Due to the limitation of the angle, we dissociated the esophagus to the inferior pulmonary vein level and merged with the laparoscope. At the same time, the abdominal operation under laparoscopy was performing (Fig. 1C).

### Flexible mediastinoscopy

**Fig. 2:**
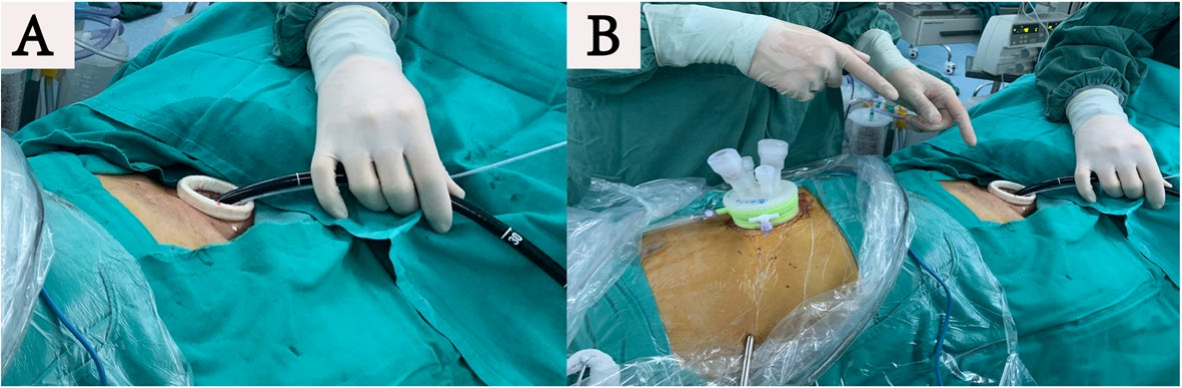
**A** Flexible mediastinoscopy and irrigation tubing entered the mediastinum through cervical incision. **B** CO2 gas was blown in to provide a stable surgical field

**Fig. 3:**
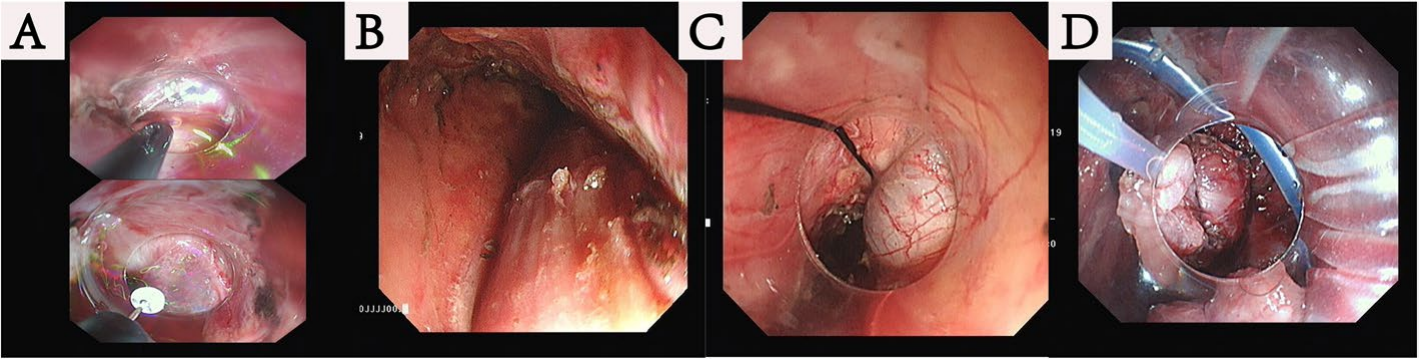
**A** The flexible mediastinoscopy utilized an IT knife and articulatory hook to dissociate the esophagus. **B** The esophagus was dissociated under the flexible mediastinoscope. **C** The distal esophagus was connected to suture for traction and extracted through subxiphoid incision. **D** Performed the gastroesophageal anastomosis in the left neck

**Fig. 4:**
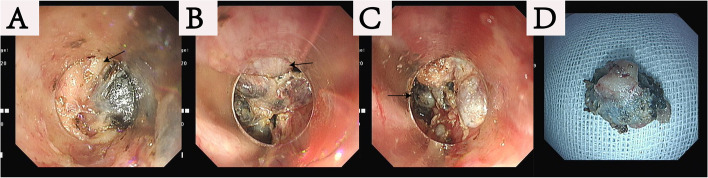
**A** Dissociated subcarinal lymph nodes (the arrow in this image indicated trachea). **B** Dissociated paraesophageal lymph nodes (the arrow in this image indicated esophagus). **C** Dissociated perivascular lymph nodes (the arrow in this image indicated the lymph node). **D** The example of removal of a single lymph node

This operation was a joint effort of the skilled endoscopists and the experienced thoracic surgeons. Made an oblique cervical incision. Bluntly dissected the cervical esophagus with fingers and opened the mediastinal cavity. The neck incision was not sealed and placed with a conventional lap-protector. A cap-based flexible mediastinoscope entered the mediastinum in this way. The neck operation required two doctors, the endoscopist performing the operation, and the assistant required to assist in the movement of the flexible endoscope (Fig. 2A). The flexible mediastinoscopy group also required CO2 insufflation (Fig. 2B). Articulatory hook or IT knife was used for the endoscopic esophageal dissection (Fig. 3A and B). A small amount of bleeding could be controlled by electrocoagulation snares. We flushed some water to provide a clear surgical field. Articulator grasper blunt dissection and IT knife were combined for lymphadenectomy (dissociated subcarinal lymph nodes, paraesophageal lymph nodes, perivascular lymph nodes, et al. (Fig. 4). Under endoscopy, we could confirm the carina, pulmonary vein, superior vena cava and lymph nodes at every station. The carbon dioxide expanded the space in the mediastinal cavity, that made the tiny structures around the aortic arch, such as nerves, bronchial arteries, and lymphatic vessels, clearly visible. The flexible mediastinoscopy dissociated downward to the diaphragmatic hiatus and converged with the laparoscope.

Routine abdominal operation under laparoscopy: dissociated the stomach and cleared away the abdominal lymph nodes. After combining the mediastinum, distal esophagus was connected to suture for traction and extracted through subxiphoid incision (Fig. 3C). Made a tubular stomach: the seromuscular layers of the lesser curvature were sutured; a 4–6 cm-wide tubular stomach was made by preservation of the gastroepiploic arcade and the right gastric artery. The isolated esophagus and stomach were drawn from the cervical incision along the esophageal bed. Performed the gastroesophageal anastomosis in the left neck (Fig. 3D). Placed a mediastinal drainage tube through subxiphoid incision. Placed the nasal feeding tube to provide the nutrition that patients needed.

### Data collection and analyses

Clinical data was collected from the medical records, and the patients were followed up for 1 month after surgery, and then every 3–6 months. Assessed perioperative variables included operation time, intraoperative blood loss, number of thoracic lymph nodes, postoperative complications (recurrent laryngeal nerve injury, chylothorax, and pleural effusion), and postoperative hospital stay.

Statistical analysis was performed by the SPSS software, version 22.0. All *p* values reported were two-sided, and *p* < 0.05 indicates statistical significance. Continuous variables were expressed as means ± standard deviation and compared by *t* test, whereas categorical variables were evaluated with Fisher’s exact test or the *χ*^2^ test.

## Results

In this study, 12 patients underwent flexible mediastinoscopy and another 17 patients underwent traditional rigid endoscopy and instruments. Postoperative follow-up data were available for all patients. The following parameters were assessed: age, sex ratio and body mass index (BMI), and tumor T staging. These parameters had no significant difference between the two groups (Table 1).

**Table 1:** Comparison of baseline demographics between the two groups (F group, flexible mediastinoscopy group; R group, rigid mediastinoscopy group)

Variables	Category	F group (*n* = 12)	R group (*n* = 17)	*P*
**Age (years)**	Range	41–78	41–75	0.46
	Mean ± SD	58.8 ± 10.3	61.9 ± 11.7	
**Gender**	Female	5	9	0.71
	Male	7	8	
**BMI (kg/m**^**2**^**)**	Range	20–29	21–29	0.32
	Mean ± SD	24.3 ± 3.1	25.4 ± 2.8	
**T staging**	Tis	6	5	0.23
	T1a	4	7	
	T1b	2	5	

All the patients underwent esophagectomy successfully. No serious complications such as tearing of azygos vein or artery and tracheal injury were observed in both groups. The operating time of the flexible mediastinoscopy group was significantly shorter than that of the traditional rigid mediastinoscopy group (192.9 ± 13.0 vs 246.8 ± 6.9 min, *P* < 0.001). As for the number of enucleated thoracic lymph nodes, flexible mediastinoscopy showed more dissected lymph nodes (8.5 ± 2.2 vs 6.0 ± 1.4, *P* < 0.01) than the traditional rigid mediastinoscopy, especially in the middle mediastinum (3.3 ± 1.0 vs 2.2 ± 0.6, *P* < 0.001) and lower mediastinum (3.1 ± 1.2 vs 1.8 ± 0.5, *P* < 0.001). There was no significant difference in intraoperative blood loss between the two groups (114.2 ± 17.5 vs 111.7 ± 16.0 ml, *P* > 0.05). In the rigid mediastinoscopy group, 1 patient had mild chylothorax and 1 patient had mild recurrent laryngeal nerve injury. However, they were cured with conservative treatment (11.8%). In the flexible mediastinoscopy group, no recurrent laryngeal nerve injury was observed. But 2 cases required catheter drainage because of postoperative pleural effusion (16.7%). No severe delayed hemorrhage, severe pulmonary infection, or anastomotic fistula was observed in the two groups. There was no significant difference in postoperative hospital stay between the two groups (7.6 ± 1.3 vs 6.9 ± 1.2 days, *P* > 0.05). All patients completed postoperative follow-up, with an average follow-up time of 11.6 ± 7.2 months. As a new technique, the flexible mediastinoscopy developed late, and the mean follow-up time was shorter than that of the rigid mediastinoscopy group (8.9 ± 3.2 vs 13.5 ± 8.5 months). No tumor recurrence was observed in either group during the follow-up period (Table 2).

**Table 2:** Comparison of clinical data between the two groups

Variables	F group (*n* = 12)	R group (*n* = 17)	*P*
**Operating details**			
Operating time (min)	192.9 ± 13.0	246.8 ± 6.9	< 0.001
Intraoperative blood loss (ml)	114.2 ± 17.5	111.7 ± 16.0	0.69
Number of thoracic lymph nodes	8.5 ± 2.2	6.0 ± 1.4	0.002
Upper mediastinum	2.1 ± 0.3	2.0 ± 0.6	0.67
Middle mediastinum	3.3 ± 1.0	2.2 ± 0.6	< 0.001
Lower mediastinum	3.1 ± 1.2	1.8 ± 0.5	< 0.001
**Postoperative complications**	16.7% vs 11.8%		
Pleural effusion	2	0	
Recurrent laryngeal nerve injury	0	1	
Chylothorax	0	1	
**Postoperative hospital stay (days)**	7.6 ± 1.3	6.9 ± 1.2	0.15
**Follow-up time (month)**	8.9 ± 3.2	13.5 ± 8.5	

## Discussion

Denise et al. [4] performed Natural Orifice Transluminal Endoscopic surgery (NOTEs) transesophageal endoscopic mediastinoscopy and thoracoscopy in swine. NOTEs technology pursues no surface incision. Numerous animal experiments have been conducted to realize this concept, and various pathways are on trial [4–7]. NOTEs is a unique emerging surgical concept that promotes the development of flexible endoscopy.

Studies have shown that flexible endoscopy can provide good vision during mediastinal exploration and can perform basic operations such as pleural biopsy. In addition, due to its flexibility, you can go wherever you want [4, 8]. Besides, it has been reported that the flexible endoscopy can create a connective tissue tunnel to the distal esophagus to perform Heller myotomy [9]. The compact connective tissue tunnel has safe propulsion and stability for the endoscope [10]. In our operation, advanced endoscopic techniques are used to assist surgical operation and replace rigid mediastinoscopy and instruments. The subxiphoid incision can indwell a mediastinal drainage tube to prevent mediastinal emphysema and serious infection to a certain extent. More advanced technology to minimize surgical trauma and alleviate patient suffering.

Flexible endoscopy may have absolute advantages in the mediastinum, such as the ability to identify avascular embryonic tissue planes and conform to the tortuous mediastinal structure. Shorter operation time and undifferentiated intraoperative blood loss can prove the advantages. The lymphadenectomy under previous animal experiments requires the endoscopic ultrasound-guidance, positioning or the support of technologies such as nano-carbon [11–14]. In our clinical application, we have enough experience in MAE. A low level of positive pressure carbon dioxide insufflation could provide ample exposure space, which is not a cumbersome operation.

The flexible mediastinoscopy can reach anywhere close enough through the endoscopic magnification. Blunt and sharp separation completes the lymphadenectomy jointly. Instead of lymph node polymer, select a single lymph node dissects. Some reports state that it is a safe strategy to dissect the left recurrent laryngeal nerve lymph nodes (RLN LNs) during esophageal cancer surgery under the flexible laparoscope [15]. Our results and experience also verify that it is safe and effective to perform this operation through articulator grasper and IT knife in the context of neuro-denseness. The transparent cap on the front can reduce the damage to the adjacent mediastinal structure theoretically and provide a more stable operating vision. In comparison, conventional mediastinoscopy requires some additional auxiliary rigid instruments to expose the surgical space. In the narrow space, it is not only necessary to overcome the triangular position of rigid instruments but also to overcome the influence of breathing movement. For rigid instruments, it is difficult even impossible to access to the distant esophagus and mediastinum. It is hard to reach the level of inferior pulmonary vein.

Due to the LCS and trachea’s nearly parallel angle and the poor visual field, it is not easy to dissociate the subcarinal lymph nodes. In contrast, since dissociating middle and lower mediastinal lymph nodes is the easier in the narrow endoscopic vision and the operational port is flexible, the flexible mediastinoscopy is more suitable.

However, the risk of postoperative pleural effusion in the flexible mediastinoscopy group was higher than that in the conventional mediastinoscopy group. Our conjecture is that endoscopists were relatively unfamiliar with the esophageal outer boundary initially, which was caused by the injury of pleura, and it is proved by the fact that the subsequent patients did not have such complications. Clear fluid drained from pleural canals, no bacterial infection found in hydrothorax culture, and no severe mediastinal infection found in postoperative CT, all of this proved that our aseptic operating environment was qualified.

So far, the indications and contraindications for the MAE are still controversial, and there is no comprehensive and recognized standard. Many comparative studies have demonstrated the feasibility of MAE, and in the course of long-term follow-up, the treatment effect is similar to transthoracic esophagectomy [2, 16, 17]. MAE avoids transthoracic operations, and it can be considered as a more friendly technique for patients with poor cardiac and pulmonary function, or a history of pleural disease [17]. It does not need to change position. Besides, the operation time is relatively shortened. Early studies reported numerous instruments and methods to improve MAE, but the standard technology has not yet established [18]. Conventional mediastinoscopy has not been widely used due to the limited vision and operational inconveniences. Besides, it is just regarded as a palliative surgical treatment for the patients with significant tumor invasion or mediastinal lymph nodes involvement. We believe that the flexible mediastinoscopy could make MAE indications more extensive to some extent. As for the advanced tumors, we are making relevant attempts.

It is essential to be familiar with the esophageal anatomy and physiology in the application of flexible endoscopic techniques. The cardiothoracic surgeon’s unfamiliarity with flexible endoscopes will undoubtedly slow the transition from research protocols to clinical applications in thoracic surgery. In our operation, the flexible mediastinoscopy is carried out with the joint efforts of particularly experienced endoscopists and under thoracic surgeons with rich MAE experience. We believe that both flexible and rigid mediastinoscopy can safely achieve the operation of the thoracic esophagus, while the flexible mediastinoscopy has better advantages due to its less aggressiveness, its stability in a narrow space, and its flexibility. It provides more possibilities for MAE. We proposed the concept of flexible mediastinoscopy. We believe that, with the development of more innovative endoscopic instruments, the flexible mediastinoscopy might have great application value.

Since the flexible mediastinoscopy is a new technique and develop late, a small number of patients were included in this study. This would inevitably result in selection bias to some extent. More clinical practice is needed to confirm the advantages of the new technique.

## Conclusions

Based on the results of this study, we unveiled a new technology, proposed the concept of flexible mediastinoscopy, and applied it to the operation of thoracic esophagus and lymphadenectomy, which proved to be a feasible technique that could be transformed into an effective tool of thoracic surgery.

## Data Availability

All data generated or analyzed during this study are included in this published article.

## Abbreviations

MAE: Mediastinoscopy-assisted esophagectomy
LCS: Laparosonic coagulating shears
BMI: Body mass index
NOTEs: Natural orifice transluminal endoscopic surgery
RLN LNs: Recurrent laryngeal nerve lymph nodes
